# Efficacy of Different Progestins in Women With Advanced Endometriosis Undergoing Controlled Ovarian Hyperstimulation for *in vitro* Fertilization-A Single-Center Non-inferiority Randomized Controlled Trial

**DOI:** 10.3389/fendo.2020.00129

**Published:** 2020-03-20

**Authors:** Haiyan Guo, Jianghui Li, Xi Shen, Yanyan Cong, Yun Wang, Ling Wu, Bin Li, Hongyuan Gao, Meng Ma, Wei Zhang, Xiaoyan Mao, Yonglun Fu, Qifeng Lyu, Weiran Chai, Yanping Kuang

**Affiliations:** Department of Assisted Reproduction, Shanghai Ninth People's Hospital, Shanghai JiaoTong University School of Medicine, Center for Specialty Strategy Research of Shanghai Jiao Tong University China Hospital Development Institute, Shanghai, China

**Keywords:** endometriosis, progestogens, *in vitro* fertilization, embryo transfer, pregnancy outcome

## Abstract

**Object:** Is it possible to use different progestins cotreatment with human menopausal gonadotrophin (hMG) in women with advanced endometriosis but normal ovulation during controlled ovarian hyperstimulation (COH) *in vitro* fertilization (IVF)? Whether different progestins treatments can be an alternative choice for women with severe endometriosis in considering IVF/ICSI treatment remains unknown?

**Design:** Non-inferiority randomized clinical trial.

**Setting:** Tertiary-care academic medical center.

**Population:** Four hundred and fifty infertile patients with severe endometriosis undergoing IVF/ICSI between May 2016 and March 2017.

**Methods:** Four hundred and fifty infertile patients with severe endometriosis undergoing IVF/ICSI were randomized to: medroxyprogesterone acetate +hMG; dydrogesterone +hMG; and progesterone +hMG. Ovulation was induced with a gonadotropin-releasing hormone agonist (GnRH-a) and chorionic gonadotropin (hCG). Viable embryos were cryopreserved for later transfer.

**Main Outcome Measures:** The primary endpoint outcome was the number of oocytes retrieved. Secondary indicators included the incidence of a premature surge in luteinizing hormone (LH), the number of viable embryos, and clinical pregnancy outcomes.

**Results:** The number of oocytes retrieved was higher in the medroxyprogesterone acetate +hMG group than the two other groups (9.3 ± 5.7 vs. 8.0 ± 4.5 vs. 7.8 ± 5.2, *P* = 0.021). LH levels were suppressed after a 6-day progestin treatment in the medroxyprogesterone acetate +hMG and dydrogesterone +hMG groups, but there was a rebound of LH values in the progesterone +hMG group. No premature LH surge and ovarian hyperstimulation syndrome (OHSS) occurred. No significant differences among the three groups were observed in fertilization and pregnancy outcomes.

**Conclusion:** It is mandatory to point out that our conclusions are valid for patients with ovarian advanced endometriosis but normal ovarian functions. These results suggest three different progestins protocols are equivalent in terms of pregnancy outcomes for women with advanced endometriosis. PPOS protocol can be an alternative choice for women with severe endometriosis and normal ovarian reserve in IVF/ICSI treatment. These methods could be tested with other populations of women with endometriosis.

**Clinical Trial Registration:** www.ClinicalTrials.gov, identifier:ChiCTR-OIN-16008529.

**Trial registration date:** 2014-05-25.

**Date of first patient enrollment:** May 2016

## Introduction

Endometriosis is a disease known to be detrimental to fertility (1–3), it is a chronic, estrogen-dependent inflammatory status, but the pathogenesis of infertility associated with endometriosis remains elusive (4, 5). Synthetic progestogens, or progestins, are believed to act as progesterone receptor agonists and have been used for more than 40 years to treat endometriosis (6). It has been reported that progestin can improve the endometriosis-associated pelvic pain via suppressing RANTES production and inhibiting inflammation in the pelvis (7). The use of progestins aims to create a low E2 environment and slow down ectopic endometrium growth (6). Progestins have few side effects, and are inexpensive. Medroxyprogesterone acetate (MPA) and dydrogesterone (duphaston) decrease aromatase transcription in mice with implanted human endometrium (8).

Progesterone (medroxyprogesterone acetate, dydrogesterone, and progesterone) is an valid choice for the prevention of premature LH surge in woman undergoing controlled ovarian hyperstimulation (COH), and the pregnancy outcomes of frozen-thawed embryo transfer (FET) illustrates that the embryos originating from progesterone co-treatment with human menopausal gonadotrophin (hMG) regimen had similar developmental potential as the short protocol (9–12). Progesterone can be an effective preventive measure for moderate/severe ovarian hyperstimulation syndrome (OHSS) in controlled ovarian stimulation in normal ovulatory women and patients with polycystic ovarian syndrome (PCOS), with the promising advantages of oral administration, user convenient, and low cost (9, 13, 14). Our previous study have shown that dydrogesterone (DYG) (15) is an appropriate oral alternative for the progesterone priming ovary stimulation (PPOS) protocol and MPA is an effective option for patients with severe endometriosis during IVF-ET (16).

There are no randomized controlled trials of different progestins (MPA, dydrogesterone, and progesterone) in advanced endometriosis women but normal ovarian function undergoing in IVF/ICSI treatments. Whether the usage of three different progestins have different clinical pregnancy outcomes and live birth rates in advanced endometriosis patients remains unknown? In order to avoid bias from interfering pathophysiological mechanisms, indication was limited to ovary endometriosis. For its possible influence on ovarian function, poor ovarian reserve itself is a confounding factor which affects pregnancy. Therefore, the included patients had normal ovarian reserve function. Therefore, this randomized non-inferiority controlled trial aimed to research the possibility of using different progestins co-treatment with hMG in women with advanced endometriosis but normal ovulation during COH in IVF.

## Materials and Methods

### Study Design

This was a prospective non-inferiority randomized controlled study that was carried out at the Department of Assisted Reproduction of the Ninth People's Hospital of Shanghai Jiaotong University's School of Medicine from May 2016 to March 2017. The study was approved by the Institutional Review Board of the Ninth People's Hospital of Shanghai. The trial was registered with the Chinese Clinical Trial Registry (ChiCTR-OIN-16008529). It was based on the Declaration of Helsinki for Medical Research. All participants provided informed consents after counseling for infertility treatments and routine IVF procedures.

### Patients

Patients planning to receive IVF/ICSI treatments were qualified to participate in the study. The inclusion criteria were: (1) advanced endometriosis as determined by laparoscopy or laparotomy, all ovarian endometriomas were stripped and peritoneal endometriosis are coagulationed. staged III-IV according to the revised American Fertility Society (AFS) classification (17); (2) ovarian endometriomas that were treated surgically by laparoscopy or laparotomy before IVF and the cyst were aspirated and confirmed as “chocolate” cysts (>3 cm) at the time of oocyte retrieval; (3) age ≤ 40 years; (4) regular menstrual cycles (25–35 days in duration) over the preceding 3 months; (5) antral follicle count (AFC) of more than 4 and <20 on menstrual cycle days 2–3; and (6) basal serum FSH concentration ≤ 10 IU/L. The exclusion criteria were: (1) basal FSH >10 IU/L or no antral follicles according to ultrasound examination; (2) diagnosis of polycystic ovarian syndrome; (3) presence of a functional ovarian cyst with E_2_ >100 pg/mL; (4) hydrosalpinx; (5) adenomyosis diagnosed by laparoscopic or laparotomy or disordered myometrial echo confirmed by ultrasound, or mild adenomyosis diagnosed by magnetic resonance imaging (MRI); (6) mild endometriosis or peritoneal endometriosis.

### Sample Size

This was a prospective non-inferiority trial. The sample size was estimated according to previous studies (9–12, 16). We assumed that the mean number of oocytes retrieved in the three groups was 10.0 oocytes (18) and the non-infertility margin was 2.0 oocytes. Assuming that the number of normal ovums obtained from women with endometriosis would be similar to that of patients with normal ovulation, the required sample size would be 130 for each group to obtain an α = 0.05 and power of 0.8 (PS power and sample size calculations, version 2.1.30). Considering the possibility of 10% dropouts, 150 women were included in each group.

### Randomization

The patients were randomly assigned to one of the three study groups in a 1:1:1 ratio. The randomized sequence was generated by the randomization program in SPSS 16.0 statistical software. The randomization process was prepared by an independent statistician. An envelope was opened after confirmation of the inclusion/exclusion factors and after signing the informed consent.

### Grouping

The patients were randomized to one of three groups: the MPA+hMG group; the dydrogesterone +hMG group; and the progesterone +hMG group.

### Ovarian Stimulation and Embryo Culture

The patients in group A were injected hMG (Anhui Feng yuan Pharmaceutical Co., China) at 150–225 IU/day and oral MPA (Beijing ZhongXin Pharmaceutical, China) 4 mg/day from day 3 of menstruation. The patients in group B were given hMG at 150–225 IU/day and dydrogesterone (Abbott Laboratories, Abbott Park, IL, USA) 20 mg/day from day 3 of menstruation. The patients in group C were given hMG at 150–225 IU/day and progesterone soft capsule (Utrogestan; Besins Healthcare, Bangkok, Thailand) at 100 mg/day from day 3 of menstruation (10).

Follicular monitoring was started on menstruation cycle day (MC) 9–11 and was performed every 2–3 days using a transvaginal ultrasound. The determination of serum FSH, LH, E_2_, and P levels and ultrasound exams were done simultaneously. The dose of hMG was adjusted according to the E_2_ levels and ovarian response. Our previous work showed that co-triggering with GnRHa 0.1 mg and a low dose of hCG (1,000 IU) had a more beneficial effect on oocyte maturation than triggering with GnRHa alone in the MPA cotreatment with gonadotropin in a general population of infertile women (9, 12). Therefore, when there were more than three dominant follicles with diameters >18 mm, oocyte maturation was induced by Decapeptyl (0.1 mg) (Ferring Pharmaceuticals Ltd., Saint-Prex, Switzerland) and hCG 1,000 IU (Lizhu Pharmaceutical Trading Co, China). Transvaginal ultrasound-guided oocyte retrieval was performed 36–37 h after triggering. Follicles were retrieved if the diameter reached 18 mm. Oocytes were fertilized using either conventional IVF or ICSI depending on the semen parameters.

### Examination of Embryo Quality

The embryo quality was assessed according to the criteria of Cummins et al. (19) and Matsuzaki and Schubert (20). The embryos were frozen by vitrification on the third day after oocyte retrieval if more than six blastomeres was detected and the degree of fragmentation wad <50%. Other embryos were cultured until the blastocyst stage for further examination, and only blastocysts with good morphology were cryopreserved.

### Transfer of Cryopreserved-Thawed Embryos

Natural FET cycles were used for women with regular menstrual cycles. If necessary, hMG was used to stimulate monofollicular growth. Patients with thin endometria during natural or stimulation cycles were recommended to adopt the hormone replacement protocol. A hormone substituted cycle was performed for patients with thin endometria during natural or stimulation cycles which had be previously described (16). The maximum number of transferred embryos was two per patient. The progesterone supplementation was continued until 10 weeks of gestation after pregnancy was achieved.

### Endpoints

The primary endpoint was the number of oocytes retrieved. The secondary endpoints were the incidence of LH surge, the number of viable embryos, and the clinical pregnancy rate. The complications during gestation (such as rupture of ovarian cyst and infection) were also used for assessment. Other indicators included the methods for delivery, fetal outcomes, birth defects, and neonatal death.

The criteria of LH surge was serum LH >15.0 mIU/ml on the trigger day (21). Information that was actively sought at the time of follow-up included hypertensive disorders developing during pregnancy or during puerperium, diagnosis of gestational diabetes, and diagnosis of placental disorders. The follow-ups during the gestation were conducted in the first, second, and third trimesters, as well as the puerperium phase. The last follow-up was conducted at the time of assessing the major defects of the neonates. Clinical pregnancy and ongoing pregnancy were considered to be the presence of a gestational sac with fetal heart activity, as assessed by ultrasound at 7 and 12 weeks of gestation, respectively. The implantation rate was defined as the number of gestational sacs divided by the number of embryos transferred. The early miscarriage rate was defined as the proportion of patients with spontaneous pregnancy termination before the gestational age of 12 weeks.

### Safety

The pre-specified safety endpoints were the percentage of women with moderate or severe ovarian hyperstimulation syndrome (OHSS). The criterion for cycle cancelation was no viable embryos for cryopreservation. All side effects were noted.

### Hormone Measurements

Serum FSH, LH, E_2_, and P were measured on day 3 of the stimulation cycle, the trigger day, and the day after trigger (12 h later after the injection of GnRHa and hCG). Hormone levels were measured by chemiluminescence (Abbott Laboratories, Abbott Park, IL, USA). The upper limit of E_2_ measurement was 5,000 pg/mL. The lower limits were: FSH = 0.06 IU/L, LH = 0.09 IU/L, E_2_ = 10 pg/mL, and *P* = 0.1 ng/mL.

### Statistical Analysis

Efficacy analyses were based on the intent-to-treat population. Normal distribution was tested using the Kolmogorov-Smirnov test. Continuous data with a normal distribution were presented as mean ± standard deviation (SD) and analyzed using ANOVA and the *post-hoc* Bonferroni test. Continuous data with a non-normal distribution were presented as median (range or interquartile range) and analyzed using the Kruskal-Wallis test and Mann-Whitney U test, as appropriate. We constructed a binary logistic regression model to quantify the related factors of profound LH suppression (LH levels on trigger day <1.0 mIU/ml) in all participants (forward stepwise method). Statistical analysis was carried out by an independent statistician using SPSS 16.0 (IBM, Armonk, NY, USA). Two-sided *P*-values were considered statistically significant.

## Results

### Patient Characteristics

**Figure 1:**
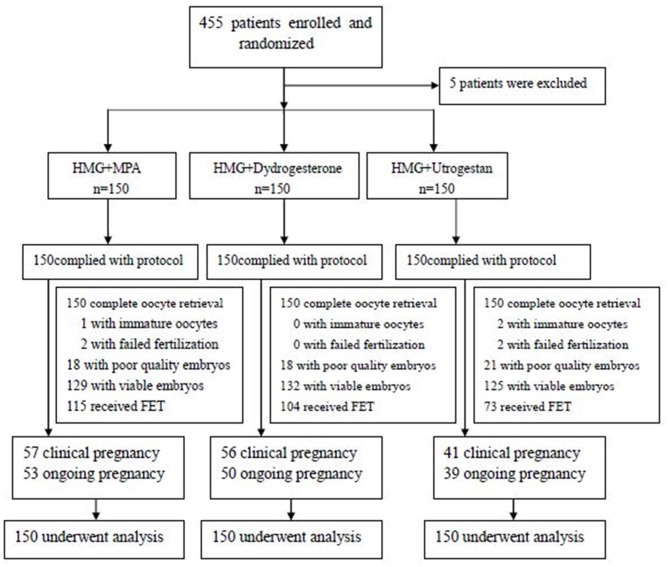
Study flowchart.

A total of 455 patients were assessed for eligibility from May 2016 to March 2017. Five patients were excluded; the remaining 450 were randomized: the MPA+hMG group underwent 150 IVF/ICSI and 115 FET cycles; the dydrogesterone +hMG group underwent 150 IVF/ICSI and 104 FET cycles; and the progesterone +hMG underwent 150 IVF/ICSI and 73 FET cycles (Figure 1).

There were no significant differences among the three groups in terms of age, BMI, AFC, duration of infertility, previous failed FET cycles, and the rate of ovarian advanced endometriomas that were treated surgically by laparoscopy or laparotomy. There were higher basal LH levels in the MPA+hMG group compared with the dydrogesterone +hMG and progesterone +hMG groups (4.14 IU/L vs. 3.48 IU/L vs. 3.24 IU/L, *P* < 0.001) (Table 1).

**Table 1:** Baseline characteristics of the women in the three groups undergoing IVF/ICSI.

**Characteristics**	**MPA+HMG**	**Dydrogesterone +HMG**	**Progesterone +HMG**	***P***
Cycle (*n*)	150	150	150	
Age, years	32.8 ± 3.4	33.0 ± 3.2	33.1 ± 3.4	0.739
BMI (kg/m^2^)	20.6 ± 2.2	21.2 ± 2.8	20.8 ± 2.3	0.157
Duration of infertility, years	3.2 ± 2.3	2.9 ± 2.2	3.2 ± 2.2	0.387
Primary subfertility	63.3 (95/150)	66.7 (100/150)	76.0 (114/150)	0.098
Parity				
0	96.0 (143/150)	94.0 (141/150)	96.7 (145/150)	0.549
1	4.0 (7/150)	6.0 (9/150)	3.3 (5/150)	0.549
Previous failed FET cycles				
0	84.0 (126/150)	84.0 (126/150)	77.3 (116/150)	0.225
1	4.0 (6/150)	7.3 (11/150)	10.7 (16/150)	0.073
≥2	12.0 (18/150)	8.7 (13/150)	12.0 (18/150)	0.564
Initial treatment				
IVF	68.7 (103/150)	71.3 (107/150)	70.7 (106/150)	0.871
ICSI	26.7 (40/150)	24.0 (36/150)	24.7 (37/150)	0.868
Antral follicle count (AFC)	10.4 ± 3.9	10.6 ± 4.5	10.2 ± 3.8	0.184
Basal FSH, IU/L	6.11 ± 1.46	5.93 ± 1.49	6.12 ± 1.84	0.521
Basal LH, IU/L	4.14 ± 1.79^a^^b^	3.48 ± 1.66^a^	3.24 ± 1.50^b^	<0.001
Basal E2, pg/mL	39.53 ± 16.04	39.11 ± 15.14	40.00 ± 19.30	0.684
Basal P, ng/mL	0.29 ± 0.11	0.31 ± 0.14	0.34 ± 0.52	0.479
Rate of endometriomas removal, %	42.0 (63/150)	36.0 (54/150)	32.7 (49/150)	0.237

*BMI, body mass index; FSH, follicle stimulating hormone; LH, luteinizing hormone; E_2_, estrogen; P, progesterone*.

a *P < 0.05 MPA+hMG vs. Dydrogesterone +hMG*.

b *P < 0.05 MPA+hMG vs. Progesterone +hMG*.

### Hormone Profiles During Treatment

**Figure 2:**
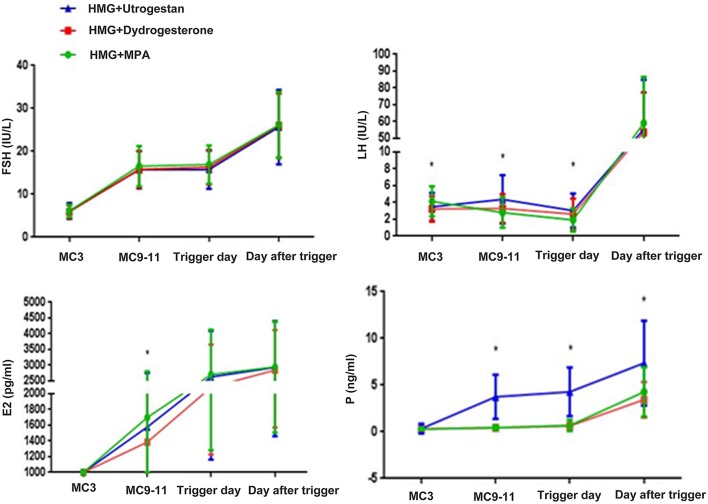
Hormone profiles in the three groups. The mean ± SD values show the temporal associations among circulating concentrations of follicle-stimulating hormone (FSH), luteinizing hormone (LH), estrogen (E_2_), and progesterone (P). The green line shows the MPA+HMG group, the red line refers to the dydrogesterone +HMG group, and the blue line refers to the progesterone +HMG group. *P < 0.05 at the time point.

FSH levels were significantly higher after hMG administration and were steady during ovarian stimulation. After triggering, the average FSH levels were similar among the three groups. The basal levels of LH in the MPA+hMG group was higher than in the other two groups (MPA+hMG: 4.14 ± 1.79 IU/L; dydrogesterone+hMG: 3.24 ± 1.50 IU/L; progesterone +hMG: 3.48 ± 1.66 IU/L; *P* < 0.001) (Figure 2).

The mean LH levels on MC9 were slightly higher compared with that on MC3 in the progesterone +hMG group (MPA+hMG: 2.75 IU/L; dydrogesterone +hMG: 3.10 IU/L; progesterone +hMG: 4.16 IU/L; *P* < 0.001), but with no LH surges being detected. values of LH on the trigger day were obviously lower in the MPA+hMG group compared with the two other groups, while LH levels on trigger day were obviously higher in the progesterone group (MPA+hMG: 1.92 IU/L; dydrogesterone +hMG: 2.61 IU/L; progesterone +hMG: 3.04 IU/L; *P* < 0.001) and then increased significantly (MPA+hMG: 59.17 IU/L; dydrogesterone +hMG: 53.58 IU/L; progesterone +hMG 55.71 IU/L; *P* > 0.05) at 10 h after trigger.

The average E2 levels on MC9 were lower in the dydrogesterone +hMG group compared with the MPA+hMG group (MPA+hMG: 1708.98 ± 1106.77 pg/mL; dydrogesterone +hMG: 1385.70 ± 991.50 pg/mL; progesterone +hMG: 1576.61 ± 1172.39 pg/mL; *P* = 0.039). The E2 levels were similar among the three groups on the trigger day and the day after the trigger.

Serum *P*-values in the progesterone +hMG group were higher than in the two other groups (MPA+hMG: 0.51 ng/mL; dydrogesterone +hMG 0.44 ng/mL; progesterone +hMG: 3.97 ng/mL; *P* < 0.05), on the trigger day (MPA+hMG: 0.68 ng/mL; dydrogesterone +hMG: 0.62 ng/mL; progesterone +hMG: 4.28 ng/mL; *P* < 0.05), and on the day after the trigger (MPA+hMG: 4.28 ng/mL; progesterone +hMG: 3.44 ng/mL; dydrogesterone +hMG: 7.33 ng/mL; *P* < 0.05).

### Ovarian Stimulation, Follicle Development, and Oocyte Performance

The number of oocytes retrieved in the MPA+hMG group was higher compared with the dydrogesterone +hMG and progesterone +hMG groups (9.3 vs. 8.0 vs. 7.6; *P* = 0.021). The number of mature oocytes retrieved in the MPA+hMG group was slightly higher compared with progesterone +hMG group, but was not different compared with the dydrogesterone +hMG group (7.8 vs. 6.9 vs. 6.5; *P* = 0.049). The numbers of top-quality embryos (2.7 vs. 2.4 vs. 2.1; *P* > 0.05) and viable embryos (3.1 vs. 2.9 vs. 2.7; *P* > 0.05) showed no significant difference among the three groups (Table 2).

**Table 2:** Stimulation and embryonic characteristics of the women in the three groups.

**Characteristics**	**MPA+HMG**	**Dydrogesterone +HMG**	**Progesterone +HMG**	***P***
HMG dose, IU	1882.5 ± 388.8^a^^b^	1756.0 ± 364.0^a^	1721.4 ± 437.5^b^	0.001
HMG duration, days	8.7 ± 1.5	8.6 ± 1.5	9.0 ± 1.8	0.076
No. of >14 mm follicles on the trigger day	7.1 ± 3.8	7.0 ± 4.4	7.2 ± 4.5	0.916
Oocyte retrieved, *n*	9.3 ± 5.7^a^^b^	8.0 ± 4.5^a^	7.8 ± 5.2^b^	0.021
D3 high-quality embryos, *n*	2.7 ± 2.4	2.4 ± 2.2	2.1 ± 2.1	0.098
Viable embryos, *n*	3.1 ± 2.5	2.9 ± 2.3	2.7 ± 2.4	0.315
Mature oocytes, *n*	7.8 ± 5.2^b^	6.9 ± 4.0	6.5 ± 4.4^b^	0.049
Abnormal oocytes, *n*	0.5 ± 1.0	0.4 ± 0.7	0.5 ± 1.0	0.464
Immature oocytes, *n*	0.8 ± 1.6	0.6 ± 1.1	0.7 ± 2.0	0.348
Mature oocyte rate, %	83.6 (1,166/1,395)	86.3 (1,034/1,198)	83.9 (966/1,151)	0.124
Fertilization rate, %	78.0 (910/1,166)	81.2 (840/1,034)	79.8 (771/966)	0.176
Cleavage rate, %	97.0 (883/910)	97.0 (815/840)	96.6 (745/771)	0.866
Viable embryo rate per oocyte retrieved, %	33.3% (464/1,395)	36.4 (436/1,198)	34.4 (396/1,151)	0.176
D3 high quality embryo rate per oocyte retrieved, %	28.9 (403/1,395)	30.1 (360/1,198)	27.4 (315/1,151)	0.243
Blastulation rate, %	18.5 (88/477)	22.2 (101/456)	23.2 (95/409)	0.181
Cancellation rate, %	14.0 (21/150)	12.0 (18/150)	16.7 (25/150)	0.342
Decreased E_2_ after trigger, %	14.4 (19/132)	9.8 (13/133)	9.2 (10/109)	0.355
Preovulation, %	0 (0/132)	0.8 (1/133)	0 (0/109)	NA
Immature oocyte rate, %	9.0 (125/1,395)	7.1 (85/1,198)	8.8 (101/1,151)	0.166
Abnormal oocyte rate, %	5.7 (80/1,395)	5.1 (61/1,198)	6.1 (68/1,124)	0.590
The rate of LH <15 after trigger	2.0 (3/150)	1.3 3 (2/150)	0 (0/150)	0.379
The rate of LH <0.1 on the trigger day	27.6 (40/145)^a^^b^	13.0 (19/146)^a^	7.0 (10/142)^b^	<0.001
Incidences of OHSS, %	0	0	0	/
Incidences of premature LH, %	0	0	0	/

a *P < 0.05 MPA+hMG vs. Dydrogesterone +hMG*.

b *P < 0.05 MPA+hMG vs. Progesterone +hMG*.

### Pregnancy Outcomes of FET Cycles

In the MPA+hMG group, 87 women completed 115 FET cycles, of which 57 women had a clinical pregnancy, and 53 women had an ongoing pregnancy (**Table 4**). In the dydrogesterone +hMG group, 78 women completed 104 FET cycles, of which 56 women had a clinical pregnancy, and 50 women had an ongoing pregnancy. In the progesterone +hMG group, 63 women completed 73 FET cycles, of which 41 women had a clinical pregnancy, and 39 women had an ongoing pregnancy. A total of 542 embryos were thawed, and the rate of viable frozen-thawed embryos was 100%. The clinical pregnancy rates per transfer (49.6% vs. 57.9% vs. 56.2%; *P* > 0.05) and implantation rates (33.8% vs. 34.2% vs. 38.9%; *P* > 0.05) were similar in the three groups. The ongoing pregnancy rates (46.1% vs. 48.1% vs. 54.4%; *P* > 0.05), the ectopic pregnancy rate, and miscarriage rate showed no significant differences. The live birth outcomes including rate of term or premature delivery, vaginal or cesarean delivery were similar in the three groups, as well as the complications (Table 3).

**Table 3:** Pregnancy outcomes of frozen-thawed embryos.

**Characteristics**	**MPA+HMG**	**Dydrogesterone +HMG**	**Progesterone +HMG**	***P***
Patients	87	78	63	
FET cycles	115	104	73	
Thawed embryos, *n*	213	190	139	
Viable embryos after thawed, *n*	213	190	139	
Transferred embryos, *n*	1.9 ± 0.4	1.8 ± 0.4	1.9 ± 0.3	0.273
Endometrial thickness of transfer day, mm	11.3 ± 1.6	11.7 ± 2.9	11.3 ± 2.2	0.762
Cleavage stage (D3 or D4)	87.0 (100/115)	83.7 (87/104)	94.9 (74/78)	0.067
Blastocyst (D5)	13.0 (15/115)	16.4 (17/104)	5.1 (4/78)	0.067
Endometrium preparation, *n* (%)				0.506
Natural cycle/mild stimulation	69.6 (80/115)	67.3 (70/104)	75.4 (55/73)	
HRT	30.4 (35/115)	32.7 (34/104)	24.7 (18/73)	
Clinical pregnancy rate per transfer, %	49.6 (57/115)	53.9 (56/104)	56.2 (41/73)	0.651
Implantation rate, %	33.8 (72/213)	34.2 (65/190)	38.9 (54/139)	0.584
Ectopic pregnancy rate, %	0 (0/57)	3.6 (2/56)	2.4 (1/41)	0.376
Single pregnancy rate, %	75.4 (43/57)	73.2 (41/56)	63.4 (26/41)	0.401
Multiple pregnancy rate, %	24.5 (14/57)	21.4 (12/56)	34.2 (14/41)	0.353
Miscarriage rate, %	7.0 (4/57)	7.1 (4/56)	2.4 (1/41)	0.556
Ongoing pregnancy rate, %	46.1 (53/115)	48.1 (50/104)	53.4 (39/73)	0.612
Mode of delivery				
Vaginal delivery rate, %	27.8 (10/36)	22.2 (6/27)	31.0 (9/29)	0.756
Cesarean delivery rate, %	72.2 (26/36)	77.8 (21/27)	69.0 (20/29)	0.957
Term delivery rate, %	88.9 (32/36)	88.9 (24/27)	69.0 (20/29)	0.064
Premature delivery rate, %	11.1 (4/36)	11.1 (3/27)	31.0 (9/29)	0.064
Birth weight (g)	3123 ± 633	3249 ± 514	3014 ± 537	0.305
Birth length (cm)	49.4 ± 3.2	49.8 ± 1.9	49.2 ± 2.9	0.743
Complications of pregnancy rate, %	11.3 (6/53)	10.0 (5/50)	10.3 (4/39)	0.974
Birth defect rate, %	0 (0/43)	0 (0/65)	0.3 (1/33)	NA

*FET, frozen-thawed embryo transfer; HRT, hormone replacement treatment*.

### Logistic Regression of LH Levels

Differences were found in the proportion of low LH value on the trigger day between the MPA+HMG and the two other groups, but not between the progesterone +HMG and the dydrogesterone +HMG groups (27.59% vs. 13.01 and 7.04%; *P* < 0.001). We performed a binary logistic regression analysis to determine the associations among the variables involved in profound pituitary suppression. The duration of infertility (OR 1.206, 95% CI 1.04–1.40, *P* = 0.013), basal FSH value (OR 0.74, 95% CI 0.41–1.14, *P* < 0.05), grouping (dydrogesterone) (OR 0.26, 95% CI 0.12–0.55, *P* < 0.01), and grouping (progesterone) (OR 0.08, 95% CI 0.03–0.23, *P* < 0.01) were independently associated with low LH levels on the trigger day (Table 4).

**Table 4:** Logistic regression of pituitary LH levels.

**Baseline parameter**	**aOR value**	**OR value**	***P*-value**	**95% CI**
Age (years)	0.947	0.978	0.665	0.886–1.080
Previous failed FET cycles	0.786	0.681	0.144	0.409–1.078
Duration of infertility (years)	1.117	1.206	0.013	1.041–1.397
Basal FSH, IU/L	0.723	0.741	0.014	0.407–1.141
Basal LH, IU/L	0.842	0.908	0.377	0.733–1.125
Basal E_2_, pg/mL	0.995	1.005	0.620	0.985–1.025
Basal P, ng/mL	1.171	1.644	0.234	0.726–3.725
HMG duration, days	1.461	1.613	0.140	0.855–3.043
AFC	1.028	0.969	0.448	0.982–1.052
Grouping (Dydrogesterone)^*^	/	0.260	<0.001	0.122–0.551
Grouping (Progesterone)^*^	/	0.083	<0.001	0.03–0.231

*CI, confidence interval; OR, odds ratio; FET: frozen-thawed embryo; FSH, follicle stimulating hormone; LH, luteinizing hormone; E_2_, estrogen; P, progesterone; AFC, antral follicle count; aOR, adjusted odds ratio*.

* *The reference group was the MPA+HMG group*.

## Discussion

The number of retrieved oocytes were higher in the MPA+hMG group than that of the progesterone +hMG group. One possible reason for this finding is associated with the excessive inhibition of the pituitary suppression, which contribute to differences in ovarian stimulation and response, resulting in an increased hMG dose and oocyte retrieved compared with the two other groups (12, 15). The extent of pituitary suppression is directly associated with the hMG dose (12). Another possible reason may be related to the slightly shorter time interval from trigger to oocyte retrieval in the progesterone +hMG group compared to the two other groups. The binary logistic regression analysis shows excessive inhibition of the pituitary suppression in MPA+hMG than the other two groups which could contribute to the higher retrieved oocytes. Pituitary LH levels were suppressed after a 6-day progestin treatment in the MPA+hMG and dydrogesterone +hMG groups, but there was a rebound of LH values in the progesterone +hMG group after 6 days of treatment at 100 mg/day, which was in line with our previous study in normal ovulatory women using progesterone (10, 11). One possible reason for this finding is associated with the extent of pituitary suppression at duration days and dose in the progesterone +hMG group during COH, because the differences of dose, absorption, metabolism, and clearance of oral P are different in different progestin groups. Our previous study had proved microparticle progesterone (Utrogestan) at 100 mg is as effective as Utrogestan at 200 mg in reducing premature LH surge during controlled ovarian hyperstimulation. No significant differences were observed in the number of mature oocytes, clinical pregnancy rate (10). Therefore, we chose 100 mg microparticle progesterone in this study. The binary logistic regression analysis in all participants showed that the low LH level on the trigger day was associated with different progestin groups, baseline FSH value, and duration of infertility. These results were different from the study by Dong et al. (21).

In the present study, hormonal measurements showed that the pituitary LH levels were suppressed after an 8-day MPA treatment, and no premature LH surge was observed during COH in either groups. Then, the LH levels increased significantly at the time of trigger 10 h later. The levels of LH on the day after trigger were similar in the three groups, suggesting that the ovaries showed normal reactions during ovarian stimulation in endometriosis patients receiving the different progestins regimen. Time-dependent effect of different progestins were found in this trial, which was in line with our previous studies (10, 11, 21). This “dose-time effect” has been observed in previous large-scale trials on the use of progestins for endometrial transformation or ovulatory inhibition (22, 23). Different progestins showed no detrimental role on oocytes and embryo development potential in normally ovulating women in our previous reports (9, 13), which is recognized (24).

Different progestins administration showed good ovum and embryo quality. Different progestins may reduce the inflammatory state generated by the metabolic activity of the ectopic endometrium, as well as the consequent immune response (25, 26). Indeed, progesterone has been shown to reduce the extent of inflammation in traumatic brain injury (25). In addition, animals study showed that progesterone can cause immunosuppression of leukocytes (26). Progestogens can inhibit the expression of NF-κB, thus reducing the expression of RANTES and different inflammatory factors (7). The application of progestogens to promote ovulation improved the inflammation and immune status of the patients with endometriosis, which could help improving the qualities of the ovums and embryos. Progestogens alone are instead generally well-tolerated, have a more limited metabolic impact than danazol or GnRH agonists, are inexpensive, and may be used on a long-term basis (27).

In the present study, one preovulation was found in dydrogesterone +hMG group. The rate of LH <15 (low ovary response) after trigger was also similar among the three groups. And subsequent study further demonstrated that triptorelin combined with hCG 1,000 IU could result in the best embryo performance in those suboptimal response (28). So, there was no need to give an rescue HCG injecting in these patients. The rate of decreased E_2_ levels after trigger compared with that on the trigger day was also similar among the three groups. No one experienced OHSS or premature LH. Therefore, the different progestin + hMG protocols were feasible for producing competent oocytes/embryos in patients with advanced endometriosis. The profound LH suppression state in the present study did not impair the quality of the oocytes and embryos. In the present study, despite the higher serum P levels in the progesterone +hMG group compared with the other two groups during COH, there was no impairment of the outcomes of oocytes end embryos.

In the multivariate analysis, the baseline FSH levels were independently associated with pituitary LH levels. No association was found with baseline LH values, which is not in agreement with previous findings (29, 30). The inhibitory effects in the progesterone and dydrogesterone groups were weaker than in the MPA group, but were nonetheless statistically significant. The three groups showed no difference in low LH rate after trigger or incidence of premature ovulation, so they were all equal in LH suppression and preventing premature LH surge. The effects were time- and dose- dependent on progestin.

A major limitation of the present study is that the calculation of sample size was made according to the incidence of number of oocyte retrieved in the MPA, dydrogesterone and progesterone protocols in normal ovulatory women. However, this limitation is overcomed when the sample size was re-estimated using the findings of the study. The number of oocytes retrieved in the MPA+hMG group was 9.3 in the study, the needed sample size is 117, consequently, the sample sufficient, so it does not affect the statistical effectiveness. Another probable bias is that we are unable to assess the nature and severity of peritoneal endometriosis in the aspiration group. So the precise stage of endometriosis remained unknown, and the standard of assessment for ovarian function in our study is AFC. AFC is not a good choice for assessing ovarian reserve in endometriosis. Endometriosis, for local inflammation, fibrosis, or adhesions caused by previous surgery lead to impaired visualization of follicles. Therefore, AMH can be used as a better ovarian reserve test. Subsequent we will supplement the patient's AMH indicator. In addition, some of the participants had not finished their FET cycle by the time of submission owing to reasons such as being ill, busy, divorced, poor uterine environment, etc. However, Because the endpoint is the number of oocytes obtained, the number of frozen transplant cycles does not affect the efficacy of our study. Finally, it is mandatory to point out that our conclusions are valid for patients with ovarian advanced endometriosis but normal ovarian functions. In this study, we compared the effects of different progestogens in IVF/ICSI, but not the effects of different regimens using for pituitary down-regulation GnRH agonist or antagonist, this could be the drawbacks of the study. This is based on the mature FET technology, and Chen et al. (31) research confirmed that FET has more advantages than fresh embryo transfer in PCOS.

## Conclusion

Therefore, for patients with endometriosis, the progesterone (medroxyprogesterone acetate, dydrogesterone, and progesterone) +hMG program may be a better option, although the number of oocytes obtained by different progestins is not the same, but there is no difference between the rate of good embryos and the pregnancy outcome. However, we will continue to study the health-economic indicators such as the effect of ovulation, the time of treatment, and the number of visits for the long-term and progesterone programs. In conclusion, the results strongly suggest that all three protocols are equivalent in terms of fertilization and pregnancy outcomes for women with advanced endometriosis but normal ovulation during COH in IVF. Additional studies are still necessary to confirm and refine these results.

## Data Availability Statement

All datasets generated for this study are included in the article/supplementary material.

## Ethics Statement

The study was approved by the Institutional Review Board of the Ninth People's Hospital of Shanghai. It was based on the Declaration of Helsinki for Medical Research. All participants provided written informed consents after counseling for infertility treatments and routine IVF procedures.

## Author Contributions

HGu, WC, and YK carried out the studies, participated in collecting data, and drafted the manuscript. XS, YW, JL, and YC performed the statistical analysis and participated in its design. LW, BL, HGa, MM, WZ, XM, YF, and QL helped to draft the manuscript. All authors read and approved the final manuscript.

### Conflict of Interest

The authors declare that the research was conducted in the absence of any commercial or financial relationships that could be construed as a potential conflict of interest.
